# Comparative evaluation of marginal gaps of lithium disilicate crowns measured using stereomicroscopy and micro‑computed tomography

**DOI:** 10.1007/s00784-026-06943-3

**Published:** 2026-05-30

**Authors:** James Dudley, Andy Min

**Affiliations:** Adelaide Dental School, The University of Adelaide, Adelaide, Australia

**Keywords:** Marginal gap, Crown, Lithium disilicate, Scanning, Milling, CAD-CAM

## Abstract

**Objectives:**

This study compared the marginal gaps of CAD-CAM lithium disilicate (LDS) crowns measured using two-dimensional (stereomicroscopy) and three-dimensional (micro-computed tomography) instruments at 12 and 360 measurement points.

**Materials and methods:**

Twenty-four typodont model lower left first molars were prepared for LDS crowns by undergraduate dental students in a dental simulation clinic. An LDS crown was constructed for each preparation using a Trios 3 scanner / Sirona inLab MC X5 milling unit at a cement space of 100 μm. Each crown was placed onto its corresponding original tooth preparation, and the marginal gap was measured using stereomicroscopy and micro-computed tomography (micro-CT) at 12 and 360 selected measurement points. The mean marginal gap (MMG) was calculated using each measurement method.

**Results:**

The MMG measured by stereomicroscopy was 82.54 μm with 12 measurement points and 85.74 μm with 360 measurement points. For micro-CT, the MMG was 96.56 μm with 12 points and 89.83 μm with 360 points. A two-way repeated-measures ANOVA demonstrated no significant main effect of measurement instrument (F₁,₂₃ = 0.35, *p* = 0.56, ηp² = 0.015) and no significant main effect of measurement density (F₁,₂₃ = 1.24, *p* = 0.28, ηp² = 0.051). There was no significant interaction between measurement instrument and measurement density (F₁,₂₃ = 0.87, p = 0.36, ηp² = 0.036).

**Conclusions:**

No statistically significant differences in MMG measurements were detected between stereomicroscopy and micro‑CT for LDS crowns under the present in vitro conditions. The number of measurements made did not significantly influence the marginal gap values obtained using either measurement instrument.

**Clinical relevance:**

The marginal accuracy of LDS restorations is critical for long-term clinical success. Assessing in vitro crown marginal gap using stereomicroscopy and micro-computed tomography provided comparable marginal gap measurements irrespective of the number of measurement points. Extending marginal gap measurements to in vivo studies is essential to substantiate clinical observations.

## Introduction

Lithium disilicate (LDS) ceramic crowns are widely used within restorative dentistry due to their lifelike aesthetics and ability to protect compromised teeth [1]. Computer-aided design and computer-aided manufacturing (CAD-CAM) has further streamlined their fabrication, making LDS crowns more routine in clinical practice [2]. A key determinant of their long-term success is the marginal adaptation. Excessive marginal discrepancies create ecological niches for oral bacteria adherence and proliferation, increasing the risk of periodontal inflammation, carious lesions, dentinal hypersensitivity and endodontic inflammation, ultimately compromising restoration longevity [3–5].

Historically, inconsistencies in terminology and measurement methods have created ambiguity in defining the marginal adaptation. In 1989, Holmes et al. devised a clear description pertaining to the marginal adaptation of a restoration, referred to as the marginal gap (MG) [6]. The MG is defined as the perpendicular distance measured from the internal surface of a crown casting to the axial wall marginal of the tooth preparation [6].

The threshold for a clinically acceptable MG has been contested. The widely cited guideline originating from McLean and von Fraunhofer’s clinical observations in 1971 [7] has been adopted in subsequent literature as a reference point. However, the reference of 120 μm serves as a practical benchmark rather than an absolute clinical standard. Ensuing in vitro studies suggested stricter limits (< 90 μm) [8, 9] with more broadly reported values for ceramic crowns ranging from 7.5 μm to 206.3 μm [10–12]. A recent umbrella analysis of systematic reviews of in vitro studies evaluating MG’s proposed a contemporary acceptable limit for in vitro crown MG of 120 μm, agreeing with that historically proposed as a clinical guideline [13].

The instruments for measuring MG’s are broadly classified as two-dimensional (2D) or three-dimensional (3D) and destructive (DE) or non-destructive (ND). The most employed instruments include direct view microscopy (2D, ND), scanning electron microscopy (3D, ND), impression replica (2D, ND), cross-sectioning (2D, DE), micro-computed tomography (3D, ND), 3D superimposition (3D, ND) and virtual fit (3D, ND). Each instrument presents unique advantages and inherent limitations that must be considered during selection. In vitro MG assessment has frequently used direct view methods such as stereomicroscopy which provide an affordable, reproducible and non-destructive method of assessment [14, 15]. However, stereomicroscopy is restricted to two-dimensional analysis and requires multiple images for a comprehensive circumferential assessment, increasing the likelihood of operator and projection errors [10, 15, 16]. More recently, micro-computed tomography (micro-CT) has gained popularity as another non-destructive alternative and is capable of generating volumetric three-dimensional renderings for assessment [17].

A further consideration is the minimum number of MG measurements required for an accurate assessment. Previous studies have indicated the selection of measurement points has often been driven by convenience rather than robust scientific rationale [14]. A large range of measurement points has been reported for single ceramic crowns, ranging from 2 to 240, with a mean of 34 measurements [14]. In recently conducted comparable research, 12 measurement points at four predetermined locations was used primarily for practical convenience [13, 14, 18, 19]. The efficiency of these methods may overlook localised margin discrepancies when compared to high-density circumferential measurement. It has also been suggested at least 50 measurements per crown is required; however this recommendation was based on calculating arithmetic means from a limited sample size aimed at minimizing measurement error rather than establishing an evidence-based standard [20]. Currently, there is no consensus on the optimal number of measurements required to yield accurate and clinically meaningful MG measurements.

The aim of this study was to compare the MG’s of CAD-CAM LDS crowns measured using stereomicroscopy (two-dimensional) and micro-CT (three-dimensional) instruments at 12 and 360 measurement points. The null hypotheses were:

1. There is no significant difference in the MMG of CAD-CAM LDS crowns using stereomicroscopy and micro-CT measurement instruments.
2. There is no significant difference in the MMG of CAD-CAM LDS crowns using 12 and 360 selected measurement points.

## Materials and methods

The study was performed at The University of Adelaide and due to its in vitro nature involving typodont models, formal ethics approval was not required under institutional policy.

Crown preparation.

Twenty-four typodont model (Columbia, Columbia Dentoform, Long Island City, NY, USA) lower left first molars were prepared for LDS crowns by undergraduate dental students in a dental simulation clinic as part of a previous study [21]. The crown preparations followed standardised pre-clinical teaching parameters to ensure clinical suitability for a full coverage LDS crown (IPS E.Max CAD, Ivoclar Vivadent, Schaan, Liechtenstein) [21].

LDS crown construction.

Each crown preparation was scanned using a Trios 3 (3Shape, Copenhagen, Denmark) scanner [19]. The corresponding LDS crown was designed by an experienced dental technician using Sirona inLab CAD Software Digital Design (version 22, Dentsply Sirona, Bensheim, Germany) and sequentially milled from an IPS e.max CAD, shade LT A2, size C14 LDS block using an inLab MC X5 (version 22, Sirona, Dentsply Sirona, Australia) milling unit at a predetermined radial and occlusal cement space of 100 μm and marginal ramp width of 50 μm [19]. Crowns were numbered from 1 to 24. New burs were inserted into the MCX5 milling unit at the commencement of milling and replaced after every eight crowns.

**Fig. 1:**
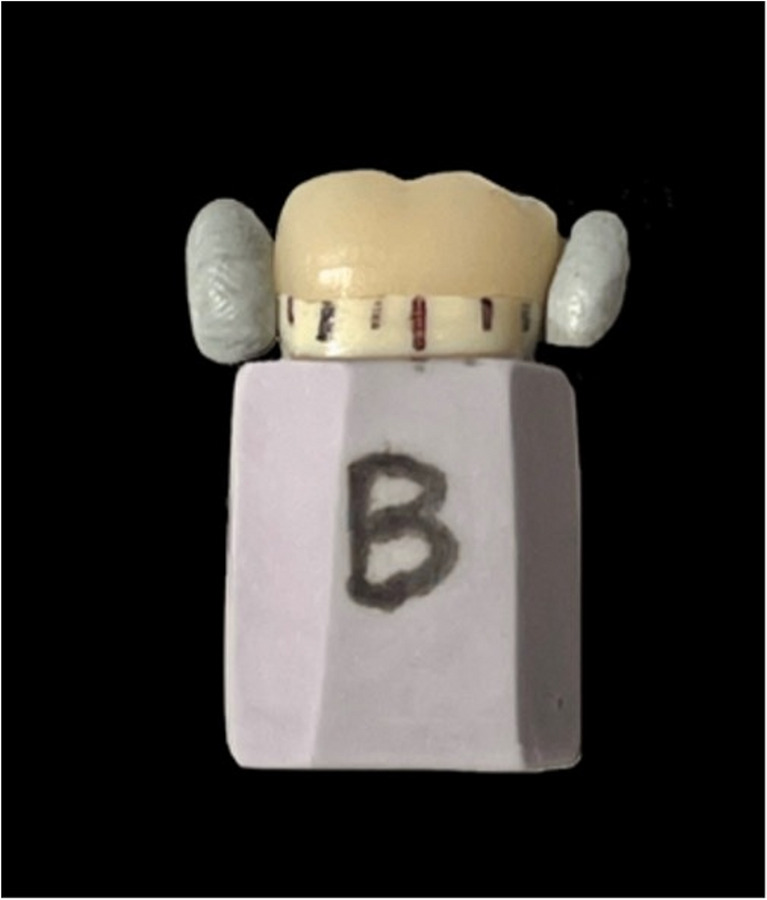
LDS crown seated on tooth die stabilised with removable adhesive, mounted onto a PVS base, showing central reference and orientation markings

**Fig. 2:**
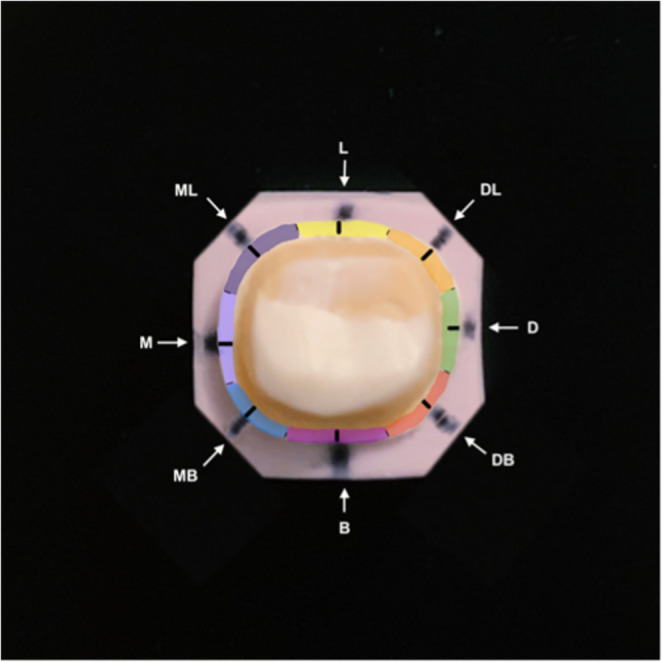
PVS base with central line markings and highlighted eight predetermined segmentations

Crowns were desprued and subjected to crystallization in a Programat P500/G2 (Ivoclar Vivadent, Australia) furnace without application of glazing, staining or any adjustment. Each LDS crown was seated onto its corresponding crown preparation and secured with a removable adhesive (Blu‑Tack, Bostik Australia) retained within a polyvinylsiloxane (PVS) rectangular base (Fig. 1). The rectangular PVS bases were cut to retain eight sides and a line marked in the centre of each face to identify the measurement segment: buccal (B), disto-buccal (DB), distal (D), disto-lingual (DL), lingual (L), mesio-lingual (ML), mesial (M), mesio-buccal (MB) (Fig. 2). Each central line marking was flanked by two boundary lines defining the segment’s borders (Fig. 1). Crowns were measured in consecutive order as initially numbered.

### Number of measurements

To assess the effect of measurement density on marginal gap evaluation, two predefined numbers of measurement points were employed.

For the 12‑point analysis, four representative anatomical surfaces (buccal, distal, lingual, and mesial) were selected, as these surfaces are commonly used in marginal gap assessment and capture directional variability associated with tooth morphology and crown preparation geometry. At each surface, the central reference line of the corresponding segment was used to guide the acquisition of three localised marginal gap measurements. Multiple measurements per surface were obtained to reduce the influence of localised margin irregularities and to provide limited within‑surface redundancy without disproportionately increasing measurement burden, resulting in a total of 12 measurements per crown. This configuration reflects a pragmatic balance between anatomical coverage and measurement feasibility. Importantly, the 12 point measurements were not selected as a numerical subset of the 360 points, but were independently localised using the central reference line of the corresponding segments at four anatomically representative surfaces.

For the 360 point analysis, the marginal circumference was divided into eight predefined segments (buccal, disto-buccal, distal, disto-lingual, lingual, mesio-lingual, mesial, and mesio-buccal), each bounded by lateral reference lines, and evenly spaced measurements were generated within each segment to yield 360 marginal gap values per crown. This high-density sampling approach was intended to minimise spatial undersampling and provide a circumferential reference that approximates a continuous marginal profile, rather than to represent a clinically prescribed measurement standard.

### Stereomicroscopy imaging − 360 point measurement

**Fig. 3:**
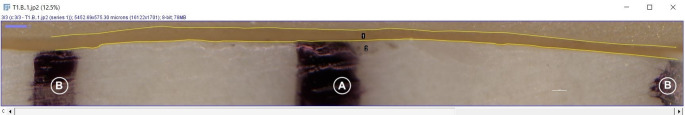
Segmented stereomicroscopy scan in ImageJ with manually drawn polylines defining the ROI for MG analysis (**A**, central line marking; **B**, segmented boundary lines)

Two-dimensional (MG) measurements were obtained using an optical stereomicroscope (Nikon SMZ25, Nikon Instruments Inc., Melville, NY USA). Each crown was secured to the adjustable stage plate using removable adhesive and orientated perpendicular to the optical axis by iterative visual alignment under standardised illumination from the built-in lighting system. Perpendicularity was confirmed by adjusting the angulation until both the crown margin and the preparation finish line appeared simultaneously in sharp focus under magnification, ensuring that marginal gap measurements were obtained normal to the marginal interface. Each segment was imaged using a 0.5x objective lens at a magnification of 13x zoom in NIS-Elements using the ‘Grab Large Free’ function. Images were calibrated to a 0.3882 × 0.3382 pixel-to-micron ratio. Two polylines were manually placed to demarcate the margins of the LDS crown and crown preparation (Fig. 3). Within Fiji ImageJ (National Institutes of Health, Bethesda, MD, USA) a custom macro script automated the measurement process within the defined region of interest (ROI). Each of the eight predetermined segments were bounded by two boundary lines (labelled ‘B’ in Fig. 3) spaced at 45° intervals, which defined the start and end of each ROI. A central line marking was positioned within each segment to represent the anatomical midpoint. Within each ROI, 45 evenly spaced vertical line measurements were generated yielding 360 vertical MG measurements per crown.

### Micro-CT − 360 point measurement

**Fig. 4:**
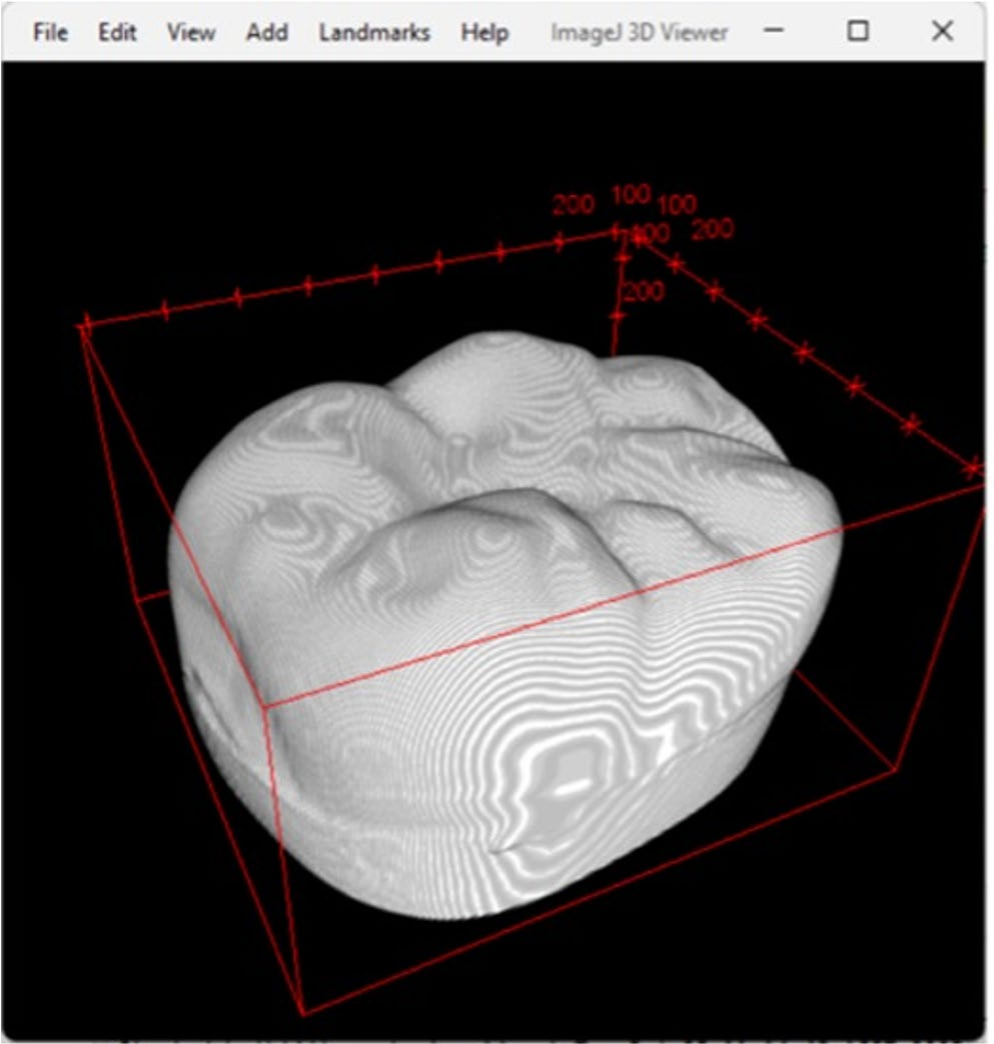
Three-dimensional rendering of the LDS crown from micro-CT

**Fig. 5:**
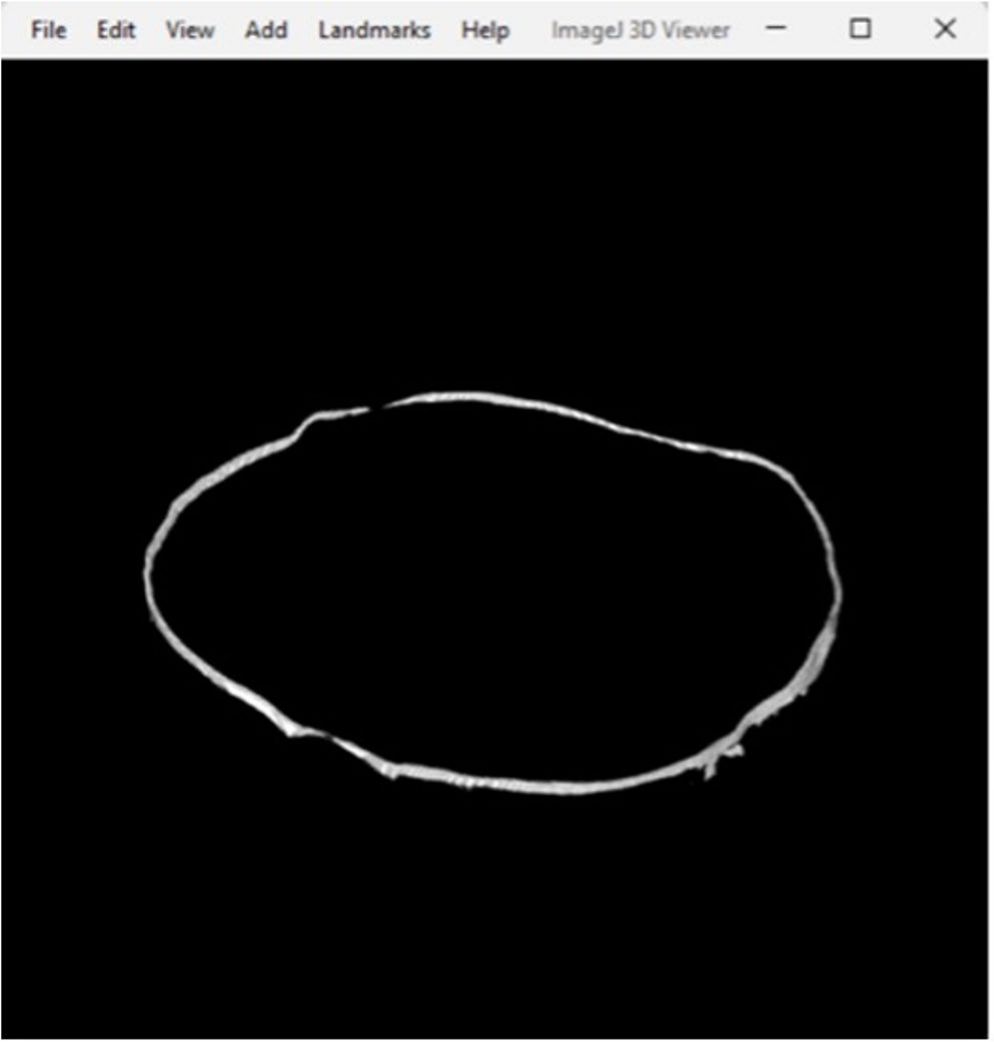
Three-dimensional rendering of the cylindrical shell for MG measurements

Three-dimensional MG gap measurements were obtained using high-resolution micro-CT (Skyscan 1276 CMOS, Bruker Biospin). LDS crowns were processed in groups of six, mounted vertically in a 40 mm holder with adhesive tape to reduce movement artifacts. Scans were processed at a resolution of 13.20 μm per voxel; ring artefact correct = 6; minimum value = 0; maximum value = 0.05; beam hardening correction = 30%. Z-stacks were aligned trans-axially using DataViewer (Bruker MicroCT, Kontich, Belgium) with further cropping of the Z-stacks completed in Fiji ImageJ. Each segmented Z-stack underwent an ilastik pixel (ilastik.org) classification process and a Gaussian blur filter was applied at a threshold of 0.5–1.0 pixels allowing for the removal and suppression of high-frequency background noise whilst preserving the integrity of the desired structural edges. Manual mask corrections were applied to aid with the refinement of the aligned MG before applying a final custom macro to close and fill the MG. A C1 channel was created from the Z-stacks with a blur threshold of 0.4–1.0 in Fiji Image J. Each MG ROI was then processed through another custom macro that produced an inverted marginal rendering set at five pixels from the predetermined MG with final fine tuning of the MG pixels completed manually. A final macro which overlaid the whole tooth mask, C1 channel and gap segmentation files for each corresponding tooth sample provided a three-dimensional rendering of the crown-tooth and final cylindrical shell of the MG used for measurements (Figs. 4 and 5). Within the custom macro, 360 vertical MG measurements were plotted in each cylindrical shell will all measurements automatically exported into Microsoft Excel (Microsoft Excel, Microsoft Corp, Redmond, WA, USA) yielding the mean, standard deviation and distribution values of each data set.

### Stereomicroscopy − 12 point measurement

Using previously obtained MG scan segments, 12 localised MG measurements were recorded for each crown at four predetermined sites: buccal (B), distal (D), lingual (lingual) and M (mesial) (Fig. 2). At each site, a centre line marking was identified from the segmented scan dataset and guided three localised MG measurements.

### Micro-CT − 12 point measurement

**Fig. 6:**
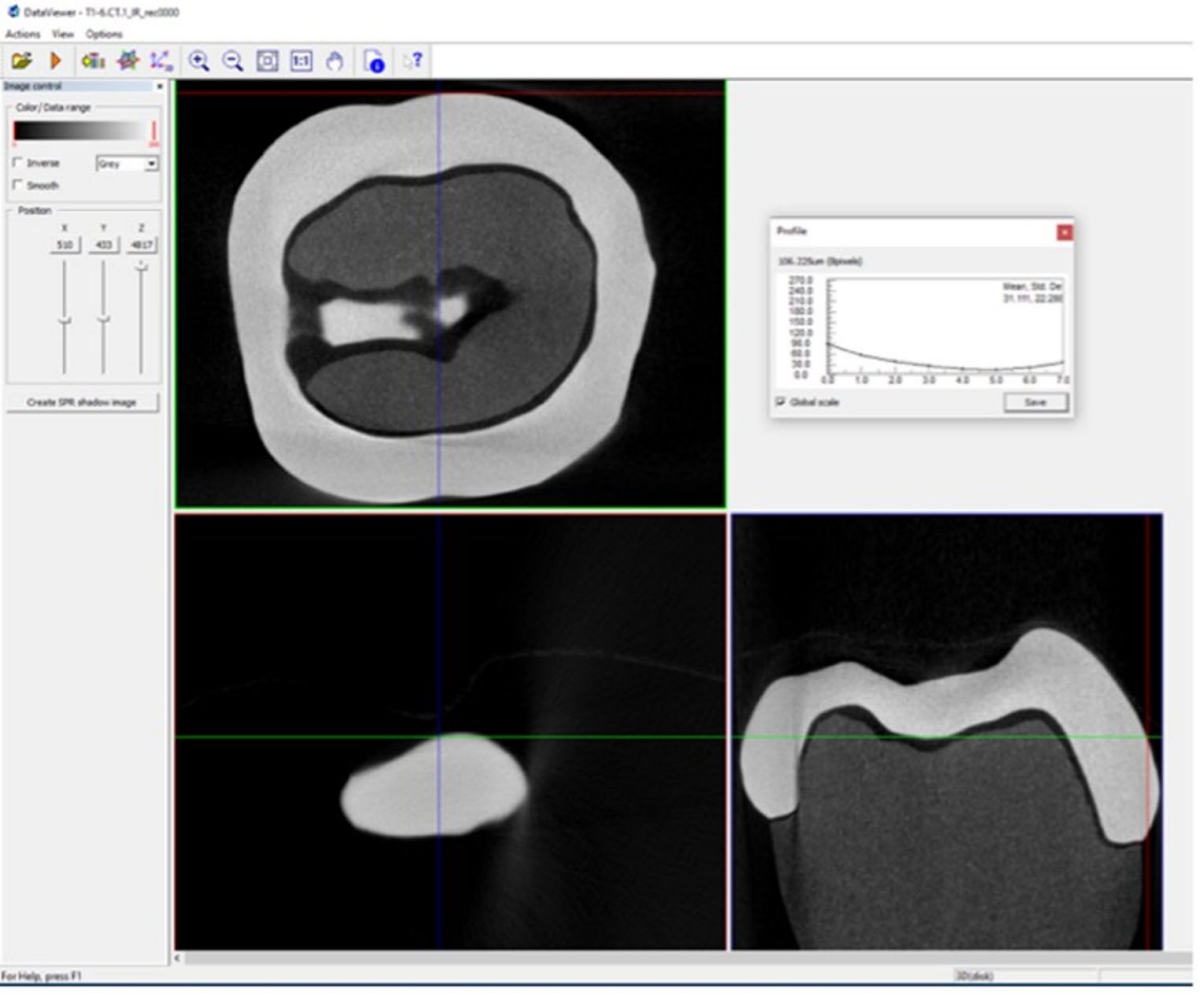
Micro-CT 12 point analysis on DataViewer

The collected z-stack images at the four predetermined surfaces (B, D, L, M) were imported into DataViewer. MG measurements were standardised by consistently orienting each crown in the same anatomical position within the scanning software as loaded in the scanning bed, with the long axis of the tooth aligned vertically. Each sample was visualised in the x, y and z-axes to ensure accurate identification and standardisation of the measurement locations and landmarks (Fig. 6). At each site, three localised MG measurements were collected resulting in a total of 12 measurements per crown.

### Measurement standardisation

A single researcher trained by an experienced supervisor conducted all measurements to minimise inter-examiner variability and potential measurement errors [22]. Measurement protocols were standardised across both stereomicroscopy and micro-CT measurement instruments, including initial calibration to reference measurements before commencing each crown. Identical points of reference and landmarks were used for each sample.

### Statistical analysis

For each measurement method, the MMG for each crown was calculated by averaging all measurements for all tooth surfaces. Quantitative data were recorded in Microsoft Excel including the mean, standard deviation, minimum, maximum, median and data distribution. A two-way repeated-measures ANOVA was conducted to assess the effects of the measurement instruments and measurement density on the MMG (α = 0.05), as well as their interaction. Normality of residuals was assessed visually using Q–Q plots, and sphericity assumptions were not applicable due to the two‑level within‑subject factors. Effect sizes are reported as partial eta squared (ηp²). Statistical significance was set at α = 0.05. A post-hoc sensitivity power analysis was performed (80% power. α = 0.05). This indicated the detectable minimum mean differences of 19.6 μm for instrument comparison, 7.5 μm for stereomicroscopy (12 and 360) and 10.9 μm for micro-CT (12 and 360).

Mann-Kendall tests were conducted to assess potential trends of MMG changes with sequential crown milling and measurement. All analyses were conducted using IBM SPSS Statistics version 26 (IBM, New York, USA) with the level of statistical significance set at *p* = 0.05.

## Results

**Fig. 7:**
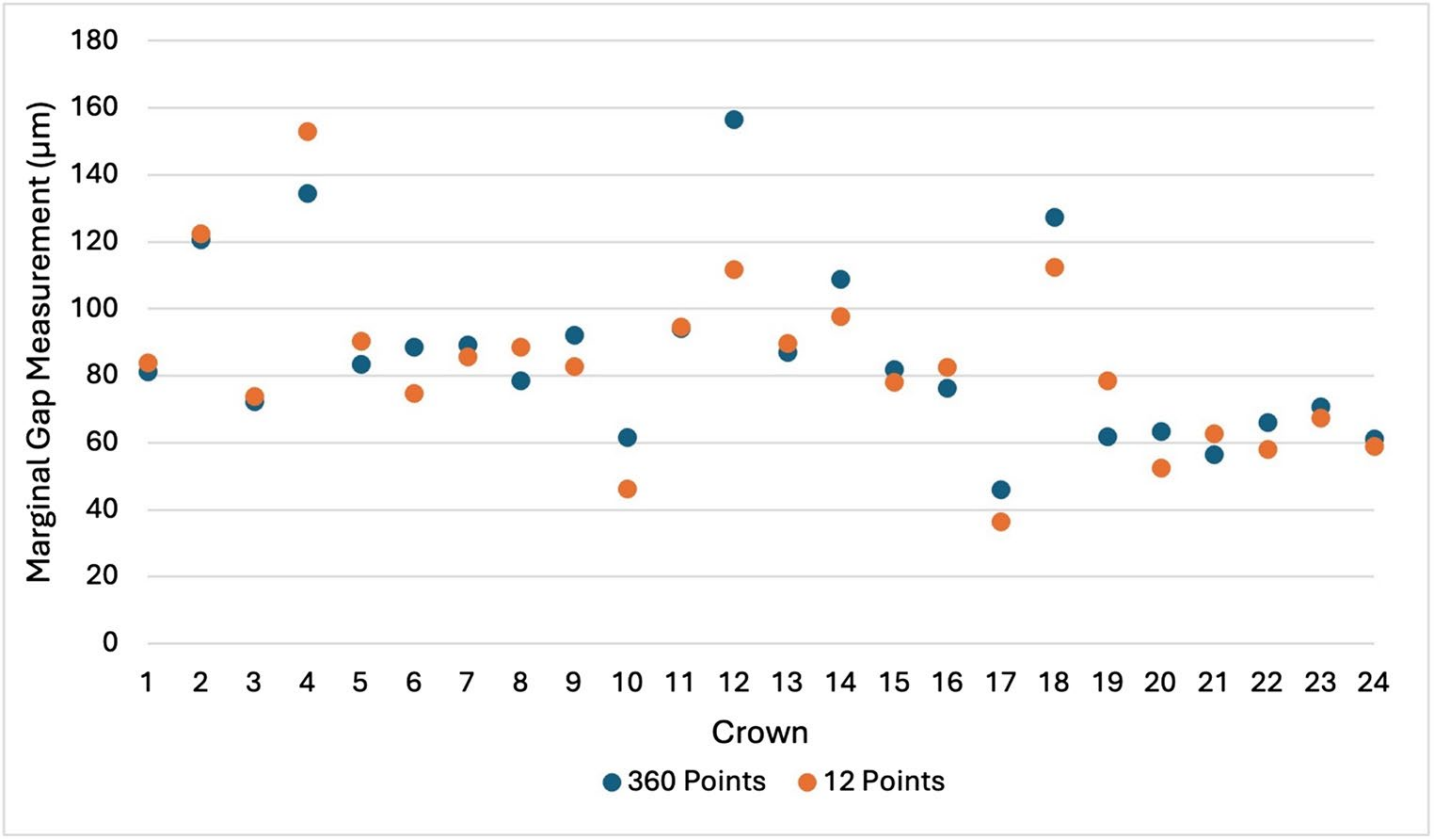
MMG measurements using stereomicroscopy at 12 and 360 measurement points (*n* = 24)

**Fig. 8:**
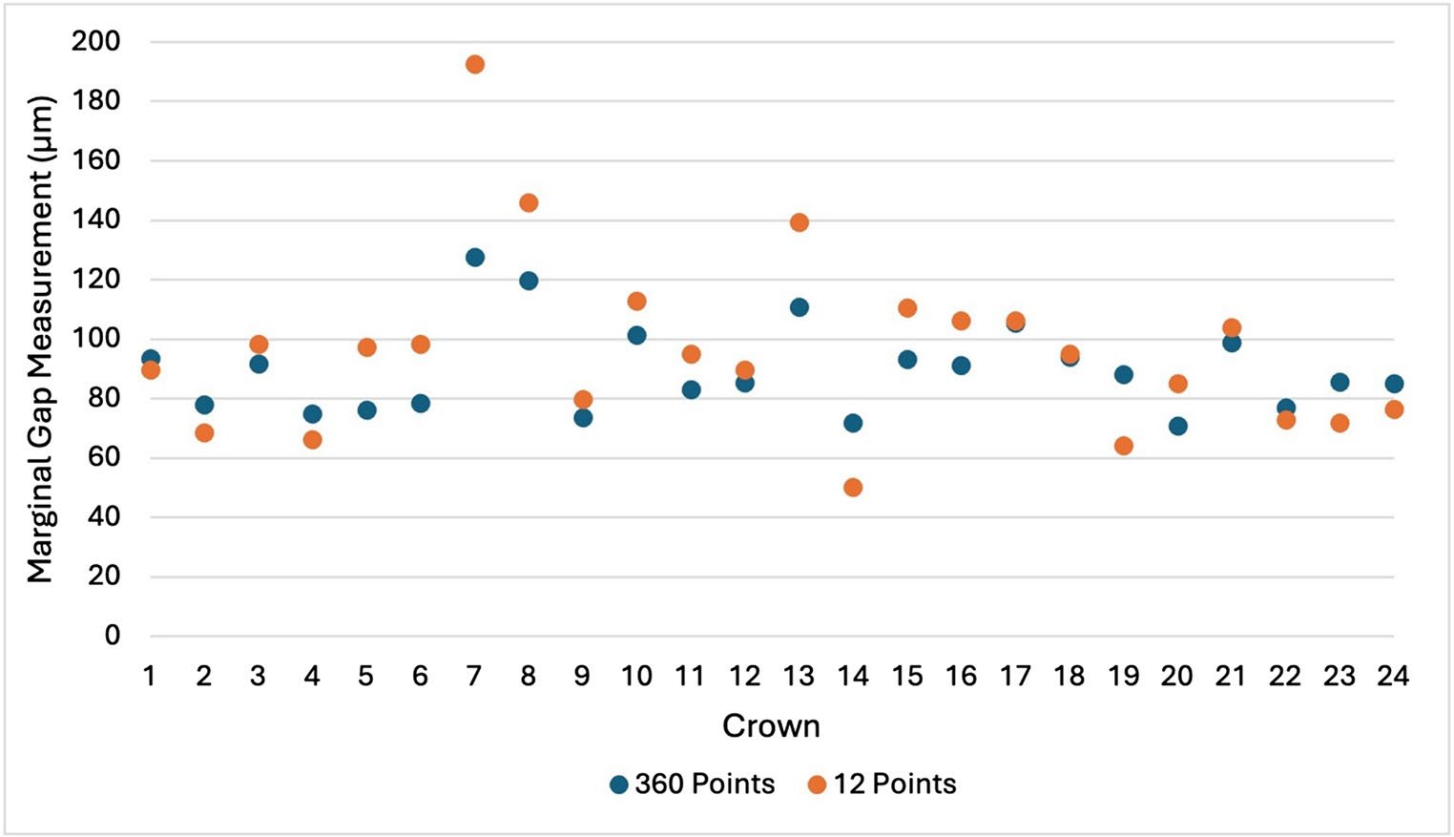
MMG measurements using micro-CT at 12 and 360 measurement points (*n* = 24)

The MG measurement data is presented in Table 1. The MMG measured by stereomicroscopy was 82.54 μm with 12 measurement points and 85.74 μm with 360 measurement points. For micro-CT, the MMG was 96.56 μm with 12 points and 89.83 μm with 360 points. The MMG measurements for each crown using stereomicroscopy and micro-CT instruments at 12 and 360 measurement points are provided in Figs. 7 and 8. Both datasets were characterised by outliers.

**Table 1:** Marginal gap measurement data (*n* = 24)

Measurement instrument	Marginal gap measurements per crown	Marginal gap (µm)				
		Mean ± SD	Minimum	Maximum	Median	95th percentile
Micro-CT	12	96.56 ± 30.34	50.33	192.54	95.12	145.06
Micro-CT	360	89.83 ± 14.97	70.93	127.58	86.88	118.45
Stereomicroscopy	12	82.54 ± 26.65	36.35	152.85	82.66	145.06
Stereomicroscopy	360	85.74 ± 26.92	45.89	156.40	81.47	133.25

Although the stereomicroscopy instrument produced smaller MMG values compared with micro-CT using both 12 and 360 selected measurement points, a two-way repeated-measures ANOVA demonstrated no significant main effect of measurement instrument (F₁,₂₃ = 0.35, *p* = 0.56, ηp² = 0.015), no significant main effect of measurement density (F₁,₂₃ = 1.24, *p* = 0.28, ηp² = 0.051), and no significant interaction between measurement instrument and measurement density (F₁,₂₃ = 0.87, *p* = 0.36, ηp² = 0.036). The effect sizes for all factors were small.

Ten of the 96 marginal gap datasets from the four measurement conditions (24 crowns x two measurement instruments x two measurement densities) exceeded the contemporary acceptable MMG limit of 120 μm [13]. Six were identified using stereomicroscopy and four using micro-CT. None of these corresponded across the two measurement instruments.

A statistically significant trend of decreasing MMG values was observed over the course of measuring the 24 LDS crowns with the stereomicroscopy instrument using both 12 and 360 measurement points, but not with micro-CT (Table 2), where the S statistic indicated the overall trend and the negative z-score indicated a decreasing trend over time.

**Table 2:** Trend analysis using Mann-Kendall tests

Measurement instrument	Marginal gap measurements per crown	S statistic	Z-score	*p*-value	Trend interpretation
Stereomicroscopy	360	−88	−2.158	0.031	Decreasing (significant)
Micro-CT	360	−8	−0.174	0.862	No significant trend
Stereomicroscopy	12	−88	−2.158	0.031	Decreasing (significant)
Micro-CT	12	−41	−0.9922	0.321	No significant trend

## Discussion

This study found no statistically significant differences in MMG measurements between stereomicroscopy and micro‑CT for LDS crowns under the present in vitro conditions.

The number of measurements made (12 or 360) did not significantly affect the MG values using either measurement instrument. Therefore, the null hypotheses were accepted. The MMG values obtained using the four different measurement methods (82.54 μm, 85.74 μm, 96.56 μm, and 89.83 μm) were slightly higher than the mean MMG of 78.8 μm reported across 13 in vitro systematic reviews evaluating ceramic crowns using various measurement instruments, as summarized in a recent umbrella analysis [13]. These findings indicate that a widely accessible, cost-effective and low‑complexity technique such as stereomicroscopy can provide marginal gap measurements comparable to those obtained using micro‑CT, which may have potential applications in quality control settings.

### Measurement instruments

When contextualising our findings within the existing literature, it is important to compare studies using similar measurement methodologies. The two instruments used in this study represent examples of the broader range of marginal gap measurement methods available [14, 23–25] and align with evidence from a recent systematic review [14] that reported no significant differences in mean marginal gap among six commonly used measurement instruments [26]. Consistent with this, the present study found that stereomicroscopy and micro‑CT provided comparable marginal gap measurements for LDS crowns under in vitro conditions, supporting the use of non‑destructive and widely accessible techniques for marginal gap assessment.

While micro‑CT offers a highly standardised and operator‑independent measurement workflow which may reduce sources of variability, formal reproducibility comparison between instruments was beyond the scope of this study. Nevertheless, its higher operational costs, comparatively lower resolution, time consuming process, and limited chairside applicability constrain its routine use in both research and clinical environments [14, 27]. Despite being more labour intensive and prone to operator variability, stereomicroscopy provides a high-resolution direct view of the crown margin at any location without the need for specialised imaging equipment, supporting its continued application as an accessible and practical method for marginal gap assessment [27].

The minor variations observed between stereomicroscopy and micro-CT are likely attributed to the inherent differences in the instruments rather than the true differences in marginal adaptation [14, 27]. Micro-CT analysis required thresholding and segmentation processes that were heavily reliant on operator judgement and disturbances in the radiopacity of the plastic tooth die occasionally impaired accurate marginal delineation [28–30]. Nevertheless, micro-CT provided a more autonomous and comprehensive circumferential assessment with reduced operator variability [27], reflecting a more consistent approach supported by its smaller standard deviation (14.97 μm) compared with stereomicroscopy (26.92 μm).

Stereomicroscopy offered a high-resolution external assessment (0.34 μm/pixel) at various locations along the MG [27]. However, a complete circumferential assessment was limited by the crown convexity and limited field of view, necessitating imaging of eight segments stitched using the “Grab Large Free” function in NIS-Elements [10, 15, 16]. While effective, this approach was significantly more time consuming and showed greater variability consistent with the larger observed standard deviation (26.92 μm) [27]. Stereomicroscopy required more manual handling than micro-CT increasing the likelihood of operator errors when more samples are required [17]. However, the statistically insignificant difference in MMG between both measurement instruments indicates stereomicroscopy as a valid alternative for a direct view MG measurement. While acknowledging the comparative nature of this study and that conventional micro‑CT is not metrology‑grade unless calibrated reference artefacts are incorporated, metrology‑grade X‑ray CT may be considered the gold‑standard reference measurement instrument for traceable assessment of complex three‑dimensional marginal geometries.

### Number of measurements

Within both measurement instruments, only minor differences in MG measurements were observed when comparing 12 versus 360 measurement points. While measuring 12 points provided a practical approximation at four pre-determined sites, the limited sample size risked overlooking localised defects or aberrations circumferentially. A minimum of 50 measurements has been proposed to yield reliable and representative results [20] however a recent comprehensive review reported that across 17 included systematic review, the median number of marginal gap measurements performed per crown was only 12 [13]. In theory, increasing the number of measurements to, for example 360, enhances the reliability and reproducibility which is particularly important for assessing an accurate circumferential marginal gap assessment [14]. However, our study which used 360 measurement points as one-degree intervals and to facilitate comparisons, found the number of measurements per crown did not significantly affect MG values for either measurement instrument, aligning with findings from a recent umbrella analysis of systematic reviews of in vitro studies evaluating MG’s, which reported no noticeable impact from measurement frequency on MG’s [14]. Accordingly, the comparison between 12 and 360 points should be interpreted as an assessment of whether conclusions derived from commonly used low density sampling are robust when evaluated against a substantially denser circumferential reference.

The largest MMG difference (6.73 μm) was observed between micro-CT 12 and 360 measurement point techniques, although the difference was not statistically significantly different. This discrepancy may be explained by the software limitations present in DataViewer (used with the 12 measurement points) that imposed a minimum detection threshold of 50.33 μm, thus yielding a standard deviation of 30.34 μm. Contrastingly, ImageJ (used with the 360 measurement points) permitted a higher resolution leading to more consistent measurements as reflected in the standard deviation of 14.97 μm. The minimum detection threshold in DataViewer also reasons the greater MMG recorded using the micro-CT with 12 measurement points, which was 10.52 μm more than the average of the other three methods. It should be noted that the comparison between 12 and 360 point analyses is partly influenced by the software platforms used, as the 12 point micro-CT measurements were performed in DataViewer, whereas the 360 point analysis relied on ImageJ-based processing and custom macros. Consequently, the observed differences reflect a combination of measurement density and software-specific processing characteristics rather than measurement density alone.

### Clinical implications

LDS is a widely used material for single crowns due to its excellent aesthetics, biocompatibility and ease of fabrication [31]. Although multiple LDS different products are available, the market is predominantly led by IPS e.max [32]. Reported short- and medium-term survival rates range from 95 to 100%, irrespective of the manufacturing procedure, a result frequently attributed to the monolithic nature of the material [31, 32]. The historically accepted guideline for in vitro crown MG of 120 μm [7] and more recently reaffirmed [13] was met in 90% of the individual crown MMG measurements in the present study, irrespective of the measurement instrument employed. This finding is clinically significant because marginal adaptation remains a key determinant of long term restoration success, influencing factors such as cement dissolution, secondary caries, and periodontal health. Achieving this benchmark across different measurement modalities suggests the manufacturing process for LDS crowns is robust and consistent.

Of the 10 crowns with MMG values exceeding 120 μm, six measured by stereomicroscopy and four by micro-CT, none overlapped across instruments. This suggests that observed differences likely reflect instrument‑specific methodological characteristics rather than fabrication‑related errors, highlighting the importance of standardized protocols and cross‑validation in future comparative studies. Further investigation incorporating repeated measurements of the same samples would be required to determine whether these differences reflect systematic instrument‑related effects or random measurement variability. Although 90% compliance with the accepted guideline is encouraging, the clinical relevance of isolated deviations remains unclear. Future studies should assess whether the MG influences long term outcomes under functional loading and oral conditions. Ultimately, in vivo investigations are essential to validate whether the high adaptation observed in vitro translates into predictable clinical performance.

### Trend analysis

An interesting observation in the current study was the progressive decrease in MMG when measured using stereomicroscopy, a trend not mirrored by micro-CT measurements. This contrasts with previous findings [33] where sequentially milled crowns exhibited a gradual increase in MG (as measured using stereomicroscopy) due to bur blunting, followed by a reduction to a value that approximated the initial milled crowns after bur replacement. However, in the present study the observed decrease in MMG did not correspond to bur replacement after every eight crowns which suggests the trend may not be related to milling bur wear. Some plausible explanations include measurement‑related factors such as operator fatigue or visual adaptation. The manual, labour intensive nature of stereomicroscopy, combined with background pressures to maintain accuracy, could introduce subtle inconsistencies over time. Further research with larger sample sizes and more controlled measurement conditions is needed to determine whether this phenomenon reflects a true material or process effect or is primarily an artifact of the measurement method. The absence of a comparable trend in the micro‑CT data, which relied on a largely automated and operator‑independent workflow, further supports the interpretation that the observed decrease in MMG is more likely attributable to measurement‑related effects rather than milling bur wear.

### Limitations

This study employed two measurement instruments and varied the number of measurement points to provide complementary insights into marginal gap assessment. However, some limitations should be considered. The study was conducted under in vitro conditions which cannot fully replicate clinical conditions such as saliva, blood, and patient compliance [34]. The crowns were assessed without luting agents which adds further clinical considerations. Although the crown preparations were originally performed by undergraduate dental students, the relatively small sample size of 24 limits the generalisability of the findings. Operator dependent variability was present using both measurement instruments. Stereomicroscopy required precise handling to avoid projection errors, while micro-CT relied on subjective thresholding and segmentation [27]. Measurements were performed by a single operator, eliminating inter‑examiner variability but introducing potential single‑examiner bias and susceptibility to measurement‑order effects, such as fatigue or visual adaptation, particularly during prolonged stereomicroscopic assessment. Additionally, the use of mean marginal gap values per specimen may underestimate the magnitude of localised marginal discrepancies, as averaging circumferential measurements can obscure site‑specific extremes that may be relevant.

The present study did not assess method agreement using correlation or Bland–Altman analysis, as it was not designed to evaluate interchangeability or proportional bias between measurement instruments. The absence of statistically significant differences in mean values should not be interpreted as evidence of measurement interchangeability or agreement, which would require dedicated agreement‑based analytical frameworks. Future studies designed to assess reproducibility through repeated measurements of identical marginal locations would provide additional insight into instrument‑specific variability and agreement.

Although a post hoc sensitivity analysis was performed, the present study was not designed within an equivalence or non-inferiority framework with a prespecified clinically meaningful margin. Consequently, while no statistically significant differences in mean marginal gap values were detected, the sample size limits inference regarding whether smaller differences may exist between measurement instruments. The absence of statistical significance should therefore be interpreted as an absence of detectable differences of the magnitude assessed, rather than confirmation of equivalence. Future investigations incorporating a priori equivalence thresholds and larger or repeated-measurement designs would be required to determine method equivalence within clinically acceptable limits.

The methodological differences between software platforms may have influenced the outcomes. DataViewer (used for the 12 point micro-CT analysis) imposed a higher detection threshold compared with ImageJ-based processing (used for the 360 point analysis) and may therefore have contributed to inflated marginal gap values [27]. Stereomicroscopy provides a two-dimensional external assessment, whereas micro-CT offers both 2D and 3D evaluation [35]. This study did not account for the three-dimensional nature of marginal adaptation, including contour discrepancies and internal fit, which may affect MG measurements and clinical outcomes [36]. Future research should address these limitations by transitioning to in vivo investigations. Incorporating multiple operators, standardized measurement protocols, and comprehensive three-dimensional analyses will strengthen reliability.

## Conclusion

No statistically significant differences in MMG measurements were detected between stereomicroscopy and micro‑CT for LDS crowns under the present in vitro conditions. The number of measurement points did not significantly influence the MG values obtained using either measurement instrument. Minor variations between instruments were attributed to methodological differences rather than true discrepancies in crown adaptation. These findings suggest that stereomicroscopy may provide MMG measurements similar to those obtained using micro-CT under controlled in vitro conditions, however formal agreement or equivalence between methods was not assessed.

## Data Availability

The data that support the findings of this study are available from the corresponding author on reasonable request.

## Abbreviations

CAD-CAM: computer-aided design and computer-aided manufacturing
LDS: lithium disilicate
Micro-CT: micro-computed tomography
MG: marginal gap
MMG: mean marginal gap
PVS: polyvinylsiloxane
